# DUO-GAIT: A gait dataset for walking under dual-task and fatigue conditions with inertial measurement units

**DOI:** 10.1038/s41597-023-02391-w

**Published:** 2023-08-21

**Authors:** Lin Zhou, Eric Fischer, Clemens Markus Brahms, Urs Granacher, Bert Arnrich

**Affiliations:** 1 Digital Health - Connected Healthcare, Hasso Plattner Institute, University of Potsdam, Potsdam, 14482 Germany; 2 Division of Training and Movement Sciences, University of Potsdam, 14469 Potsdam, Germany; 3 Department of Sport and Sport Science, Exercise and Human Movement Science, University of Freiburg, 79102 Freiburg, Germany

**Keywords:** Neuroscience, Biomedical engineering, Computer science

## Abstract

In recent years, there has been a growing interest in developing and evaluating gait analysis algorithms based on inertial measurement unit (IMU) data, which has important implications, including sports, assessment of diseases, and rehabilitation. Multi-tasking and physical fatigue are two relevant aspects of daily life gait monitoring, but there is a lack of publicly available datasets to support the development and testing of methods using a mobile IMU setup. We present a dataset consisting of 6-minute walks under single- (only walking) and dual-task (walking while performing a cognitive task) conditions in unfatigued and fatigued states from sixteen healthy adults. Especially, nine IMUs were placed on the head, chest, lower back, wrists, legs, and feet to record under each of the above-mentioned conditions. The dataset also includes a rich set of spatio-temporal gait parameters that capture the aspects of pace, symmetry, and variability, as well as additional study-related information to support further analysis. This dataset can serve as a foundation for future research on gait monitoring in free-living environments.

## Background & Summary

Gait analysis is an essential part of mobility assessment to support the diagnosis, treatment, or rehabilitation, both for injuries and diseases. Clinical gait analyses are usually performed under controlled laboratory conditions, typically using multi-camera or instrumented walkways^1,2^. However, gait characteristics differ between daily life settings and controlled laboratory environments^3,4^. Inertial measurement unit (IMU) based methods enable mobile gait analysis in free-living environments. In recent years, there has been a growing interest in developing and evaluating gait analysis algorithms based on IMU data, which has important implications for sports science, biomechanics and rehabilitative medicine^5^.

This dataset aims to contribute to the development of methods for recognizing gait modulations in daily life settings. Daily life walking often occurs while concurrently performing additional tasks, such as walking while talking on the phone. Although walking is generally considered an isolated and automatic process, there is evidence that performing a secondary task during walking (i.e., dual-task condition) significantly changes the gait pattern, indicated by a decrease in walking speed and an increase in gait variability^6–9^. Physical fatigue is another relevant factor in daily life walking. There is evidence that muscle fatigue has a negative impact on static and dynamic balance, which increases the risk of injury and/or falls in healthy adults^10^ as well as in vulnerable populations (e.g., elderly or neurological disease patients)^11,12^. Understanding gait modulation mechanisms using dual-task and fatigue scenarios and being able to recognize the changes in gait characteristics are crucial to enabling daily life gait monitoring.

Despite the high relevance of recognizing gait modulations in dual-task settings and in fatigued conditions using mobile sensors, few datasets have been made publicly available to allow the testing and development of algorithms. Moreover, the combination and interaction of physical fatigue and secondary (cognitive) task performance is prominent in real-life settings but less investigated. Table 1 summarizes studies that collected walking data under dual-task or fatigue conditions using IMUs. Most of the datasets consist of recordings with only one or two IMUs and short durations of walking, and none of the datasets are publicly available. To fill this gap, here we present the dataset DUO-GAIT: A **Gait** Dataset for Walking under **D**ual-Task and Fatig**u**e C**o**nditions. This dataset contains recordings of 6-minute walks under single (only walking) and dual-task conditions (walking while performing a cognitive task) conditions in an unfatigued (control condition) and fatigued state from 16 healthy young adults. In particular, nine IMUs were placed on the head (HE), chest (sternum, ST), lower back (sacrum, SA), left and right wrists (LW and RW), left leg and right leg (LL and RL) as well as left and right feet (LF and RF) to record tri-axial acceleration and angular velocity. The unique multi-sensor setup opens up many possibilities to re-use this dataset. These sensor placements can be used independently or in custom combinations for typical IMU gait analysis algorithms quantifying foot movement^13,14^, arm swing^15,16^ or for full-body pose estimation^17^. In addition, the dataset also includes a rich set of spatio-temporal gait parameters calculated from the IMU data, such as stride length, speed, and their coefficients of variation and symmetry values. Apart from the gait-related data, participant demographics, physiological data such as the blood lactate concentrations and heart rate as objective indicators of fatigue, and transcripts of the responses from the cognitive task are also included in this dataset for further exploration and analysis.

**Table 1:** Summary of datasets of walking with fatigue and/or dual-task using IMUs.

Dataset	Participants	Condition	IMU Placement	Total Amount of Walking	Data Availability
^34^	49 MS patients	Fatigue induced by walking	2 IMUs on the feet	6 min	On request
^35^	15 Healthy	Fatigue induced by physical work	one IMU on the right ankle	170 min working and walking	Not available
^36^	65 MS patients	Fatigue induced by walking	2 IMUs on the feet	6 min walk test and 25-foot walk	On request
^37^	17 Healthy	Fatigue induced by squatting	2 IMUs on right shank and sternum	2 × 15.5 m	Not available
^38^	18 Older adults	Fatigue induced by walking	one IMU on the heel	1 h	Not available
^39^	11 Older adults, 14 PD patients, 9 stroke patients	Dual-task with numerical stroop test	2 IMU on the shanks	multiple times 5 m	On request
^40^	54 PD patients	Dual-task with serial subtraction	8 IMUs on both feet, shanks, wrists, chest, and posterior trunk	2 × 14 m	Not available
^41^	18 Healthy and 21 with neck pain	Dual-task with head turning	3 IMUs on the forehead, upper- and lower thoracic spine	2 × 8 m	Not available
^42^	10 Healthy, 20 frail, 11 frail with MCI	Dual-task with serial subtraction or verbal naming	one IMU on the lumbar spine	3 × 7 m	Not available
^43^	20 Healthy	Dual-task with holding water	2 IMUs on the feet	6 × 14 m	On request
^44^	384 Neurological disorder patients	Dual-task with serial subtraction or motor task	2 IMUs on the feet	3 × 20 m	Not available
This dataset	16 Healthy	Fatigue and Dual-task	9 IMUs on the head, chest, lower back, wrists, legs, and feet	4 × 6 min	Available

Most of the datasets consist of data from one or two IMUs with a small amount of recording. In contrast, our dataset consists of data from nine IMUs with four times 6-minute walks, and is publicly available.

MS: Multiple sclerosis, PD: Parkinson’s disease, MCI: mild cognitive impairment.

In summary, the presented dataset contributes to the testing and development of methods for recognizing gait modulations using a mobile IMU setup. We anticipate that this dataset will be used for future research on gait monitoring in free-living environments.

## Methods

### Study participants

Sixteen healthy adults (eight males, eight females) aged 21 to 35 years were recruited for this study. All participants were free of any neuromuscular or cardiovascular diseases and did not perform strenuous physical exercises 48 hours prior to the data collection. The Physical Activity Readiness Questionnaire (PAR-Q) was used to further determine study eligibility. Participants who answered “yes” to any of the questions (i.e., indicating limitations for performing physical exercise) were excluded from the study. The International Physical Activity Questionnaire (IPAQ, short form) was used to assess the levels of physical activity in the daily life of the participants. Table 2 summarizes participant characteristics.

**Table 2:** Participant characteristics.

Variable	Mean ± SD	Min	Max
Age (years)	27.1 ± 3.8	21	35
Body Mass (kg)	71.2 ± 12.2	54	103
Height (cm)	173.8 ± 8.6	158	190
Leg Length (cm)	83.9 ± 3.8	78	94
Activity Level*	2	1	3

*1, 2, 3 means low, medium and high activity levels in IPAQ, respectively. The median is reported instead of the mean ± SD, since data contain ordinal values. SD: standard deviation.

### Experimental design

**Fig. 1:**
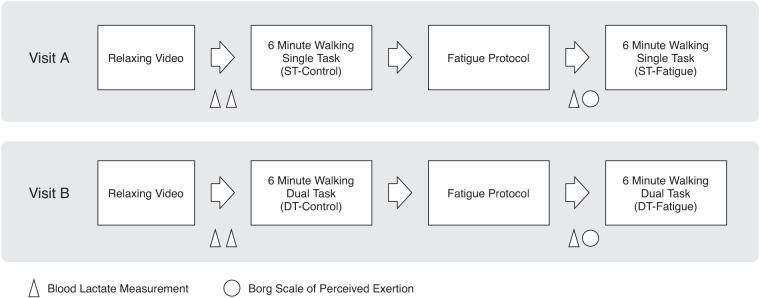
Study design. Visits A and B are randomized for each participant.

The experimental design consisted of two visits (referred to as visits A and B in the following text) that were seven days apart and randomized for each participant. To control for the effects of circadian rhythms on physical performance, the times of the two visits were less than an hour apart for the same participant. During visit A, participants watched a relaxing nature documentary for 5–10 minutes, and the experimenter measured blood lactate concentration using blood samples from the earlobe twice within an interval of 5–10 minutes. Subsequently, the participants performed two 6-minute walking sessions before and after a muscle fatigue protocol. During the 6-minute walking sessions, the participants were asked to walk at their self-selected walking speed up and down a hallway with a 35 m one-way distance. During the fatigue protocol, the participants wore a weighted vest matched to 30% of their body mass, and repeatedly stood up from a chair and sat back down until they were not able to continue. The task was performed at a self-selected, fast pace. Immediately after the fatigue protocol, blood lactate concentration was measured again, and the participants reported their perceived fatigue level on the Borg Rating of Perceived Exertion (RPE) scale (referred to as “Borg scale” in the following text)^18,19^. The procedure during visit B was identical to that of visit A, except that while walking, the participants performed a secondary cognitive task which involved the continuous subtraction of seven from a random 4-digit starting number (between 3000 and 9000) provided by the experimenter. Participants had to speak out the numbers so that we were able to record and analyze their responses. To reduce learning effects, participants practiced the dual-task 6-minute walk one time before the actual data recording. In total, four walking sessions were recorded for each participant: single-task control (ST-Control), single-task fatigue (ST-Fatigue), dual-task control (DT-Control), and dual-task fatigue (DT-Fatigue). Figure 1 provides an overview of the study design.

### Recording devices

**Fig. 2:**
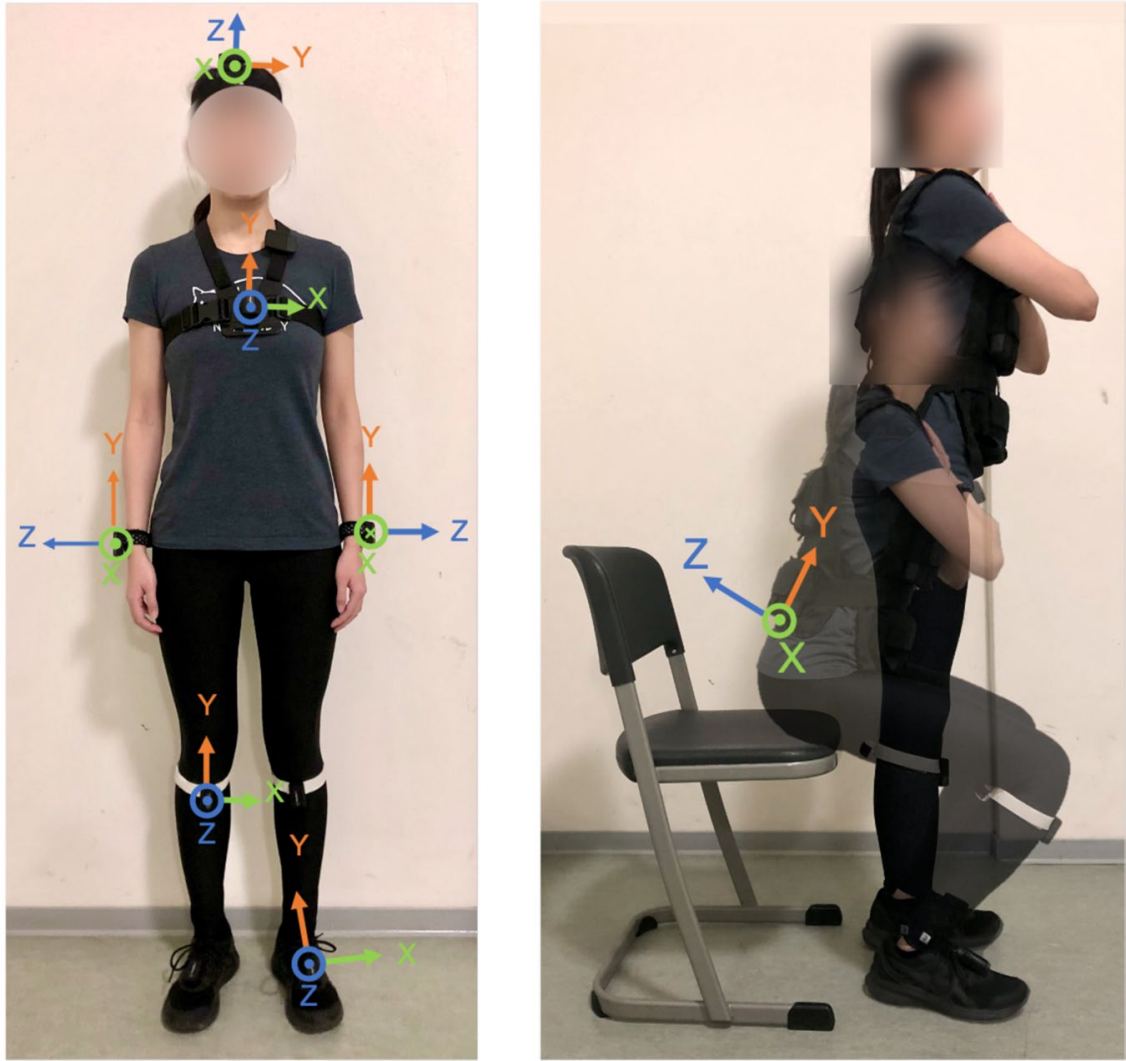
Experimental setup. Left: sensor placements and orientations. Right: sit-to-stand fatigue protocol.

Nine IMU devices (Physilog^®^5, Gait Up, Switzerland) were attached to the head (HE), chest (sternum, ST), lower back (sacrum, SA), left and right wrists (LW and RW), left and right legs (LL and RL) as well as left and right feet (LF and RF) of the participants. The IMUs were synchronized before the start of recording. Tri-axial acceleration (range: ±16 g) and angular velocity (range: ±1000 degrees/s) data were recorded at a sampling rate of 128 Hz. A heart rate sensor (Polar H10, Polar, Finland) was attached to the chest (below the sternum) to record the heart rate. During each visit, the IMUs and the heart rate sensor continuously recorded data from the start of the first walking session until the end of the second walking session. The chest IMU was removed during the fatigue protocol to allow proper positioning of the weighted vest. An audio recorder was attached close to the left collar bone for both visits A and B, and recorded responses from the number subtraction task during visit B (dual-task condition). Figure 2 shows the experimental setup.

The study was approved by the ethics committee of the University of Potsdam (63/2020) and all experimental procedures were in accordance with the latest revision of the Declaration of Helsinki. All participants provided written consent prior to the data collection.

### Data processing

For each visit, the IMU signals were segmented into three parts: the 6-minute walk under control conditions (ST-Control or DT-control), the fatigue protocol and the 6-minute walk under fatigued conditions (ST-Fatigue or DT-Fatigue) by visual examination of the IMU signals. Spatio-temporal gait parameters were extracted from walking segments using an algorithm that has been validated in previous studies^13,14^. Briefly, the algorithm takes tri-axial acceleration and angular velocity data as input, uses an error-state Kalman filter, which utilizes zero-velocity update to track errors in the sensor signal during stance periods of the foot, and estimates the 3D movement trajectory of the foot. Foot-off and initial contact events are identified using features from the angular velocity data. Temporal parameters, such as stride time and stance time, are calculated directly from the gait events. Spatial parameters, such as stride length and clearance, are calculated by segmenting the 3D foot trajectories using the gait events. Subsequently, outlier strides were identified and excluded from further analyses using the following steps: turning strides at the ends of the walkway were identified using a manual threshold on the change of foot orientation, acceleration, and deceleration strides were identified as two strides before and after the turning strides, interrupted strides (when the participants were disturbed during the 6-minute walk) were excluded using manually documented timestamps. Additional outlier strides were identified using a z-score threshold at three and excluded for further analyses. The stride-by-stride gait parameters from each participant and each foot were then aggregated into mean and coefficient of variation (CV, defined as the ratio between standard deviation and mean). In addition, gait parameters from both feet of each participant were aggregated into mean, CV, and symmetry index (SI). The symmetry index is defined as in Eq. 1, where *X*_*LF*_ and *X*_*RF*_ are the mean gait parameters of the left and right foot, respectively.:

1 $$SI=\frac{\left|{X}_{LF}-{X}_{RF}\right|}{0.5\ast \left({X}_{LF}+{X}_{RF}\right)}$$

## Data Records

**Fig. 3:**
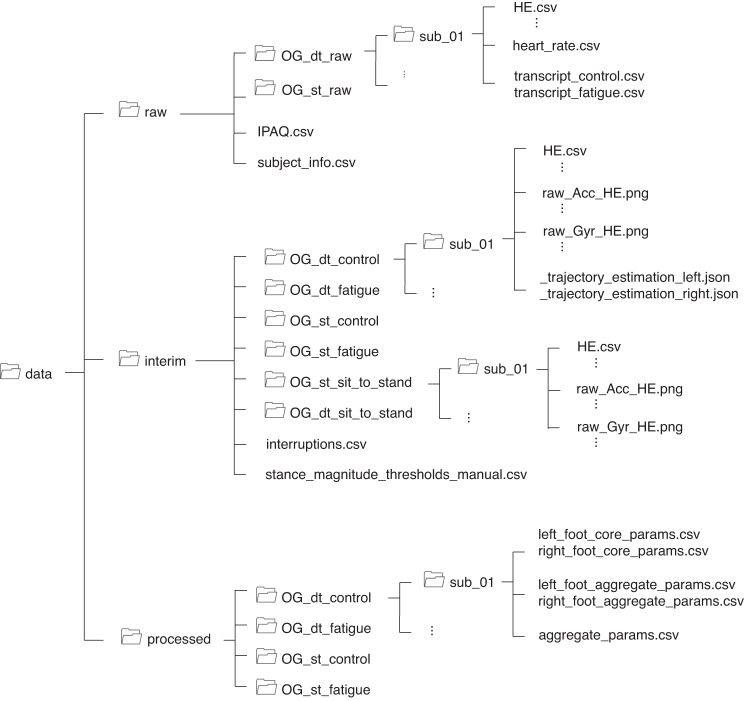
Data Structure. The dataset has three top-level folders: “raw,” “interim,” and “processed”. The “raw” folder contains continuous recordings of the entire walking sessions from the IMU devices and the heart rate sensor, as well as additional information such as cognitive task transcripts, IPAQ questionnaire responses, and demographics. The “interim” folder contains IMU data segmented into individual 6-minute walking sessions and the sit-to-stand fatigue exercise. The “processed” folder contains spatio-temporal gait parameters calculated from the feet IMU data.

The dataset can be downloaded at the Zenodo platform^20^. The dataset is divided into three top-level folders “raw”, “interim”, and “processed” as illustrated in Fig. 3. The “raw” folder contains the raw IMU recordings, heart rate recordings, transcripts of responses from the number subtraction cognitive task, responses from the IPAQ questionnaire, and demographic and anthropological information. Raw IMU data and heart rate data were continuously recorded from the start of the first walking session to the end of the second walking session, resulting in one recording for each visit and each participant. Therefore, the “OG_st_raw” folder contains the data from the entire single task visits (i.e., ST-Control and ST-Fatigue) and the “OG_dt_raw” folder contains the data from the entire dual task visits (i.e., DT-Control and DT-Fatigue) for each participant. The IMU data were saved in.csv format, which was extracted from the original binary (.BIN) format using the Physilog RTK software. Heart rate data and transcripts of cognitive task responses were saved in.csv format. Due to technical issues, heart rate data from two recording sessions (out of 32 recording sessions for all participants and visits) are not available.

The “interim” folder includes IMU data that were manually segmented into the two walking sessions and the sit-to-stand fatigue protocol for each visit by visual inspection of raw IMU signals. For quality control, the segmented accelerometer and gyroscope data of each sensor was plotted, resulting in 18 plots per participant. In addition, during the first execution of gait parameter extraction, calculated 3D feet trajectories were cached in the “interim” folder, so that for future executions, the cached trajectories can be loaded directly, sparing the computational efforts for re-calculation. The file “interruptions.csv” documents time periods where the walk was briefly interrupted, for example, when a second person enters the walking area. The strides during the interrupted time periods were marked as outliers and removed from subsequent analyses. The file “stance_magnitude_thresholds_manual.csv” documents the angular velocity thresholds used to identify stance phases for the gait analysis algorithm for each participant. The thresholds were determined by visual observation of the angular velocity signals.

The “processed” folder contains stride-by-stride spatio-temporal gait parameters extracted for each of the four walking conditions, and aggregated gait parameters in terms of coefficients of variation and symmetry for all walking conditions for each participant.

## Technical Validation

### Technical validation of the IMU data

In our previous study, we performed technical validation on the raw data quality by comparing seven different IMU models^13^. The IMU model Physilog^®^5 exhibited the best overall data quality and was therefore selected for creating the present dataset. Our other previous study also validated the gait analysis algorithm used to extract the spatio-temporal gait parameters for this dataset using two independent reference systems^21^. The results demonstrate the high quality of both the IMU raw data and the extracted gait parameters, with root mean square error of 0.05 m for stride length and 0.02 s for stride time.

### Effectiveness of fatigue and dual-task protocols

**Fig. 4:**
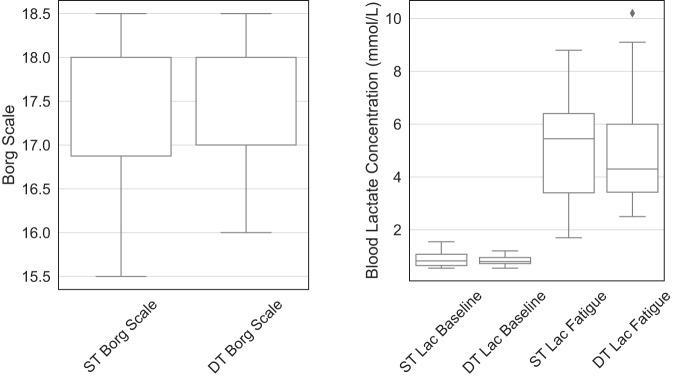
All participants reached sufficient levels of fatigue, as confirmed by Borg Rating of Perceived Exertion Scale (left) and blood lactate level (right). Fatigue levels after the sit-to-stand protocol are comparable for both single-task and dual-task visits. ST: single-task, DT: dual-task, Lac: blood lactate concentration.

The Borg scale and blood lactate measurements both confirm that all participants were fatigued after performing the sit-to-stand protocol, as illustrated in Fig. 4. All participants reported ratings of perceived exertion (RPE) values larger than 15.5 on the Borg scale. For all participants, the average blood lactate concentrations at baseline (averaged across two measurements for each person and visit) were below 2 mmol/L, which is in agreement with previously reported levels at rest^22^. After the fatigue protocol, the blood lactate concentration increased significantly for all participants, indicating muscle fatigue^23^. There were no significant differences between ST and DT conditions for both Borg scale (p = 0.76) and blood lactate level after the fatigue protocol (p = 0.92), indicating that the fatigue levels during the two visits are comparable.

Dual-task costs (DTC, %) were used to assess the effects of dual-task walking on gait characteristics^24^. DTC describes the change of a gait parameter between single- and dual-task walking, and is defined as in Eq. 2:

2 $$DTC=\frac{{X}_{ST}-{X}_{DT}}{{X}_{ST}}\ast 100$$

where X is the gait parameter of interest, *X*_*ST*_ and *X*_*DT*_ represent the parameter (averaged value of the left and right foot) under the single-task and dual-task walking conditions, respectively. Dual-task costs of example gait parameters are summarized in Table 4.

### Statistical summary of raw IMU data

To validate the completeness of the collected data, we present statistical parameters for the acceleration and angular velocity (gyroscope) data as an example. Since all IMUs are synchronized and the recordings are started and stopped simultaneously, the recording duration is identical among all IMUs in the same recording session. Table 3 summarizes the statistical parameters for different recording segments. The recordings “ST Full Recording” and “DT Full Recording” are the original non-segmented recordings from the entire single-task or dual-task visits, which include the baseline 6-minute walk (non-fatigued condition), the sit-to-stand fatigue protocol, and the 6-minute walk in a fatigued state. The recordings “ST-Control”, “ST-Fatigue”, “DT-Control” and “DT-Fatigue” are recording segments of only the 6-minute walks under the respective conditions. The recording durations of these recordings are all above six minutes, confirming that all participants completed the 6-minute walking sessions and the data was complete. The recordings “ST Fatigue Protocol” and “DT Fatigue Protocol” are recording segments of only the sit-to-stand fatigue protocol. The amount of data indicates that all participants performed the exercise, and the effectiveness of the exercise in inducing physical fatigue is confirmed by results in the section “Effectiveness of Fatigue and Dual-task Protocols”. The magnitude of acceleration (Acc. Magnitude) and angular velocity (Gyro. Magnitude) are also within the expected range for walking and squat-like exercises for the respective sensor placements. In addition, all raw IMU signals have been visually inspected to ensure data quality.

**Table 3:** Statistical summary of IMU raw data from different recording segments with left foot (LF) and sacrum (SA) sensors as examples.

IMU Placement	Recording	Recording Duration (s)	Acc. Magnitude (m/s)	Gyro. Magnitude (deg/s)
LF	ST-Control	380.97 ± 3.81	16.64 ± 1.12	199.03 ± 19.65
LF	DT-Control	384.54 ± 10.72	15.52 ± 0.77	180.47 ± 16.04
LF	ST-Fatigue	382.63 ± 2.61	16.93 ± 0.99	200.12 ± 18.42
LF	DT-Fatigue	381.23 ± 4.99	15.79 ± 0.79	183.26 ± 16.06
LF	ST Fatigue Protocol	812.49 ± 440.11	9.7 ± 0.04	10.47 ± 2.75
LF	DT Fatigue Protocol	895.23 ± 661.78	9.68 ± 0.04	10.63 ± 2.46
LF	ST Full Recording	2628.67 ± 509.79	11.92 ± 0.51	69.27 ± 12.76
LF	DT Full Recording	2840.06 ± 726.96	11.5 ± 0.39	61.92 ± 9.69
SA	ST-Control	380.97 ± 3.81	10.14 ± 0.11	47.6 ± 9.24
SA	DT-Control	384.54 ± 10.72	10.1 ± 0.09	43.83 ± 10.97
SA	ST-Fatigue	382.63 ± 2.61	10.2 ± 0.13	53.62 ± 10.77
SA	DT-Fatigue	381.23 ± 4.99	10.16 ± 0.12	53.41 ± 12.13
SA	ST Fatigue Protocol	812.49 ± 440.11	9.9 ± 0.10	36.73 ± 10.03
SA	DT Fatigue Protocol	895.23 ± 661.78	9.94 ± 0.12	36.56 ± 10.48
SA	ST Full Recording	2628.67 ± 509.79	9.98 ± 0.06	32.67 ± 5.29
SA	DT Full Recording	2840.06 ± 726.96	9.97 ± 0.05	31.33 ± 4.35

All values are expressed as mean ± standard deviations per person. The recordings “ST Full Recording” and “DT Full Recording” are the original non-segmented recordings from the entire single-task or dual-task visits. The recordings “ST-Control”, “ST-Fatigue”, “DT-Control” and “DT-Fatigue” are recording segments of only the 6-minute walks under the respective conditions. The recordings “ST Fatigue Protocol” and “DT Fatigue Protocol” are recording segments of only the sit-to-stand fatigue protocol. Acc.: acceleration, Gyro.: angular velocity measured by the gyroscope.

### Statistical summary of gait parameters

Since all participants successfully completed the 6-minute walk sessions, the amount of data is balanced for all participants and the four walking conditions. In total, seven temporal and spatial stride-by-stride gait parameters (stride length, minimum clearance, maximum clearance, stride time, stance time, swing time and stance ratio) and 27 aggregated parameters (mean, coefficient of variation and symmetry index for speed, cadence, stride length, minimum clearance, maximum clearance, stride time, stance time, swing time and stance ratio) were calculated from the IMU data. In studies investigating the effects of fatigue or dual-task walking on gait performance, stride length and walking speed are among the most reported gait parameters^8,11^. As an example, we summarized these parameters from our dataset. The mean stride length of 1.32 m to 1.44 m and the mean speed of 1.15 m/s to 1.30 m/s are within the normal range for healthy young adults reported in other studies^25^. Moreover, the algorithm used to calculate these gait parameters has been validated against gold standard reference systems in previous studies^13^. Table 4 summarizes the total number of strides, stride length, speed in each walking session, and dual-task costs under control or fatigue conditions.

**Table 4:** Statistical summary of example gait parameters.

Walking Session	Num. of Strides*	Stride Length (m)	Speed (m/s)	Stride Length DT Cost (%)	Speed DT Cost (%)
ST-Control	601.19 ± 39.39	1.44 ± 0.11	1.30 ± 0.13	—	—
DT-Control	580.19 ± 36.29	1.35 ± 0.10	1.16 ± 0.08	6.06 ± 5.56	10.47 ± 8.43
ST-Fatigue	604.00 ± 38.24	1.41 ± 0.12	1.29 ± 0.13	—	—
DT-Fatigue	579.75 ± 33.02	1.32 ± 0.11	1.15 ± 0.08	5.83 ± 5.90	10.23 ± 8.40

*Number of valid strides per person. ST = Single Task, DT = Dual Task. Summary of gait parameters are expressed as mean standard deviation.

In addition, using stride length and speed as an example, we performed two-way repeated measures ANOVA to investigate the ability of these gait parameters to distinguish walking under fatigue and unfatigued states, as well as under single- and dual-task conditions. The main effect of fatigue is significant (p < 0.05) for stride length (F(1,15) = 6.62, p = 0.02, *η*^2^ = 0.01), but not for speed (F(1,15) = 0.70, p = 0.41, *η*^2^ = 0.003). The main effect of dual-task is significant (p < 0.05) both for stride length (F(1,15) = 17.03, p = 8.95 × 10^−4^, *η*^2^ = 0.14) and speed (F(1,15) = 23.75, p = 2.05 × 10^−4^, *η*^2^ = 0.31). Detailed results of the statistical tests are summarized in Table 5. No significant interaction effects between fatigue and dual-task were found. The results indicate that the change in gait patterns induced by dual-tasking is much larger than the change induced by fatigue. Our recently published study explores this aspect in more detail and demonstrates how the effects of fatigue on gait patterns can be investigated in depth^26^.

## Usage Notes

Depending on the research question, each of the three data subsets (raw, intermediate, and processed) can be used independently or selectively combined for further analysis. The raw IMU signals from the entire recording sessions can be potentially used for developing and validating algorithms for recognizing walking bouts^27^. More generally, the data can also be used with segmentation algorithms^28^ and time-series motif identification algorithms^29^ to recognize different daily-life activities from wearable devices.

The raw IMU signals during walking can be further processed into clinically-relevant parameters for quantifying gait. Our dataset provides a rich set of data from different IMU body placements to capture the gait characteristics. Typical sensor placements used for such analyses include: using feet or lower back IMUs to extract gait parameters such as stride length, stride time, walking speed, symmetry and variation^13,14,30^, using wrist IMUs to quantify arm swing angles^15,16^, or using a sparse IMU combination (head, lower back, wrists, legs) to obtain full-body joint angles and pose estimation^17^. These clinically-relevant parameters can then be used to evaluate gait classification algorithms. Multiple IMU placements from this dataset enable identification of optimal minimized sensor setup for daily life gait monitoring^31^. In addition, the raw IMU signals during the fatigue protocol can be combined with the heart rate data, the Borg scale of perceived exertion and the blood lactate concentration to study exercise-related kinematics and its effects on fatigue levels^32^.

The gait parameters provided in this dataset can be used for developing gait classification and visualization methods for refined gait changes^33^. The demographic and anthropometrical characteristics included in the dataset (raw/subject_info.csv) help to further analyze the data. For example, to identify changes in gait patterns caused by fatigue or dual-task performance, the gait parameters could be normalized to the body height or leg length of each person.

For researchers who intend to use their custom gait analysis algorithms to extract the gait parameters for further analyses, it is important to first evaluate the quality of the extracted gait parameters. We have previously published our gait analysis pipeline along with a dataset to validate the quality of the calculated gait parameters against two independent reference systems^21^. The pipeline was built in a modular way so that new algorithms could be inserted and tested using the accompanying dataset.

## Limitations and future works

This dataset consists of data obtained from young healthy participants using nine different IMU placements to capture the whole body gait changes. These data serve as a valuable resource for exploring methods to analyze gait changes induced by physical fatigue and cognitive task performance. However, it is important to note that to increase the dataset’s usefulness for specific target groups, further studies are required to collect data from diverse populations that are more susceptible to external factors affecting their gait stability, such as the elderly or patients with neurological diseases or movement disorders. For a more comprehensive understanding of the gait changes, additional data modalities should be incorporated, including electromyography (EMG) and force data from pressure sensors. Continuous recordings of various daily life activities at home are also of interest for research on mobility-related issues.

**Table 5:** ANOVA results of example gait parameters from all participants.

Gait Parameter	Main Effect of Fatigue	Main Effect of Dual-Task
Stride Length Avg (m)	F(1,15) = 6.62, p = 0.02, *η*^2^ = 0.01	F(1,15) = 17.03, p = 8.95 × 10^−4^, *η*^2^ = 0.14
Speed Avg (m/s)	F(1,15) = 0.70, p = 0.41, *η*^2^ = 0.003	F(1,15) = 23.75, p = 2.05 × 10^−4^, *η*^2^ = 0.31

Avg = average. F = F-value, p = p-value, *η*^2^ = generalized eta squared (effect size).

## Data Availability

All data processing procedures described in this paper were performed using Python 3.7. The code repository and more detailed usage instructions can be found at https://github.com/HPI-CH/fatigue-dual-task-data. The main functionalities of the code are as follows: • Segment the IMU recordings into walking sessions and fatigue exercise • Calculate spatio-temporal gait parameters from the IMU signals • Summarize gait parameters and other study-related information
